# *Toxoplasma gondii* disrupts intestinal microbiota and host metabolism in a rat model

**DOI:** 10.1371/journal.pntd.0013768

**Published:** 2025-11-25

**Authors:** Ji-Xin Zhao, Wen-Bin Zheng, Shi-Chen Xie, He Ma, Xiao-Tong Chen, Ying-Qian Gao, Lu-Yao Tang, Meng-Ting Yang, Fu-Long Nan, Jing Jiang, Hany M. Elsheikha, Xiao-Xuan Zhang

**Affiliations:** 1 College of Life Sciences, Changchun Sci-Tech University, Shuangyang, Jilin, PR China; 2 College of Veterinary Medicine, Qingdao Agricultural University, Qingdao, Shandong, PR China; 3 Laboratory of Parasitic Diseases, College of Veterinary Medicine, Shanxi Agricultural University, Taigu, Shanxi, PR China; 4 Faculty of Medicine and Health Sciences, School of Veterinary Medicine and Science, University of Nottingham, Loughborough, United Kingdom; Zhejiang Wanli University, CHINA

## Abstract

*Toxoplasma gondii* infection disrupts the gut microbiota and host systemic metabolism, which plays a key role in the pathophysiology of toxoplasmosis. To investigate these interactions, we conducted metagenomic sequencing and untargeted serum metabolomics on 18 Sprague-Dawley rats across control, acute, and chronic stages of infection. *De novo* assembly of 148 Gb of high-quality reads produced a comprehensive non-redundant microbial gene catalog comprising over 5.7 million genes. Infection led to a marked reduction in microbial diversity and significant shifts in community structure. Chronic infection, in particular, was characterized by the enrichment of *Lactobacillus johnsonii*, *Lactobacillus intestinalis*, and *Limosilactobacillus reuteri*, alongside a marked depletion of *Akkermansia muciniphila* and *Rothia nasimurium*. These compositional changes coincided with reduced abundance of carbohydrate-active enzymes, suggesting impaired microbial metabolic capacity. Pathway analysis revealed distinct, stage- and gut-region-specific metabolic disruptions, including suppressed amino acid and energy metabolism, and enhanced glycan and carbohydrate pathways during chronic infection. Untargeted LC-MS/MS profiling uncovered 883 differentially abundant serum metabolites, enriched in pathways related to amino acid metabolism, bile acid transformation, and aromatic compound processing. Importantly, *L. johnsonii* and *L. reuteri* were positively correlated with metabolites implicated in immune modulation and oxidative stress response, whereas *A. muciniphila* showed negative associations. These findings demonstrate that *T. gondii* infection orchestrates a coordinated host–microbiota–metabolome network, advancing our understanding of disease mechanisms and pointing to novel microbial and metabolic targets for therapy.

## Author summary

*Toxoplasma gondii* is a common parasite capable of infecting both humans and animals. Although it is best known for its effects on the brain and muscles, the intestine is the first site where infection takes hold. We investigated how *T. gondii* alters the gut environment and how these changes may influence the health of the host. Using a rat model, we analyzed the intestinal microbiota and metabolites in the blood following infection. We found that *T. gondii* infection, particularly during the chronic stage, reduced microbial diversity and disrupted the balance of gut bacteria. Beneficial microbes declined, while others became more abundant, impairing the gut’s ability to break down nutrients. These microbial shifts were linked to significant changes in the host’s metabolism, including molecules related to immunity and energy regulation. Our findings highlight how parasitic infection can disturb the natural relationship between gut microbes and their host, leading to broader physiological consequences. This work provides new insights into host–microbiome–parasite interactions and may inform future strategies to prevent or treat toxoplasmosis by targeting the gut microbiome.

## Introduction

*Toxoplasma gondii* is an obligate intracellular protozoan that infects a wide range of nucleated cells and establishes chronic infections in tissues such as the brain and muscles [1]. The intestine serves as the primary entry site and a critical immunological hub during *T. gondii* infection. Following oral ingestion of tissue cysts (harboring bradyzoites) or sporulated oocysts (containing sporozoites), *T. gondii* initiates a localized intestinal phase characterized by epithelial invasion, leukocytic infiltration, and inflammation.. While infection is frequently subclinical among intermediate hosts, clinical disease is relatively uncommon in healthy adult domestic cats (*Felis catus*). Susceptibility and clinical expression vary among felid species—the definitive hosts—and, when disease occurs in cats, signs may include fever, respiratory compromise, or neurological deficits [2]. In humans, infection is usually subclinical or mild; however, immunocompromised individuals, such as those with HIV or organ transplant recipients, are at risk of severe complications, including encephalitis, ocular toxoplasmosis, and systemic disease reactivation [3]. From a public health perspective, toxoplasmosis is globally widespread and significantly contributes to congenital disorders, vision impairment, and neurological disabilities, highlighting the need for effective surveillance and preventive strategies [4]. In this context, intraepithelial lymphocytes (IELs) play a key immunoregulatory role. Particularly, TGF-β-producing IELs help suppress pro-inflammatory responses, maintain epithelial barrier integrity, and protect against severe ileitis [5].

The intestine serves as both the primary entry point for *T. gondii* and a central hub for immune activity, positioning the gut microbiome as a critical mediator in the host’s response to infection. Far beyond its role in digestion, the gut microbiota performs essential functions, including the breakdown of complex carbohydrates, vitamin synthesis, protection against pathogenic invasion, maintenance of epithelial barrier integrity, and regulation of immune development and inflammation [6–8]. When this ecosystem is disrupted, a state known as dysbiosis, broad physiological consequences can follow, including altered metabolite profiles and impaired immune function [9]. Dysbiosis has been implicated in a wide array of conditions, from infectious diseases to metabolic disorders, inflammatory bowel disease, and even neurodegeneration [10–12].

Understanding how infectious agents such as *T. gondii* reshape the gut microbiota is therefore crucial for deciphering the complex interplay between parasites, microbial communities, and host immunity. In mice, infection-induced ileitis is marked by a rapid collapse of beneficial commensals (e.g., *Lactobacillus*, *Bifidobacterium*) and a striking overgrowth of Gram-negative bacteria, particularly members of the Enterobacteriaceae (e.g., *Escherichia coli*), as well as *Bacteroides* and *Prevotella* species [13,14]. This microbial imbalance fuels severe intestinal inflammation, tissue damage, barrier dysfunction, and bacterial translocation, compounding immunopathology. Targeting this dysbiosis with antibiotics can reverse bacterial overgrowth, reduce inflammation, prevent systemic spread of microbes, and improve survival [13]. Interestingly, reintroducing Enterobacteriaceae into germ-free mice reinstates the proinflammatory response, confirming their causal role in pathology [13,14].

These findings underscore the bidirectional relationship between microbial ecology and host immune responses during *T. gondii* infection. While microbial imbalance amplifies disease severity, host immune pathways critically shape outcomes. The infection triggers an IFN-γ-dependent inflammatory loop via TLR11–MyD88 signaling, leading to Paneth cell death, microbiota disruption, and intestinal inflammation. MyD88 signaling within T cells further drives pathogenic TH1 responses [14]. Emerging evidence also points to a protective role for interleukin-22 (IL-22), a cytokine that enhances epithelial repair, induces antimicrobial peptides (e.g., Reg3β, Reg3γ), and tempers inflammation [15]. IL-22-deficient mice experience heightened epithelial damage, increased proinflammatory cytokine production (e.g., IFN-γ, IL-1β), and reduced survival, despite similar parasite loads [14]. Interestingly, germ-free conditions eliminate these differences between wild-type and IL-22-deficient mice, suggesting IL-22 acts independently of the microbiota. Supporting this, microbiota transfer from IL-22–/– mice fails to replicate the heightened susceptibility in germ-free hosts [15].

While much attention has focused on local intestinal effects, *T. gondii* infection also triggers systemic metabolic shifts, affecting lipid metabolism, amino acid balance, and energy homeostasis, through both direct resource exploitation and immune-driven metabolic reprogramming [16,17]. These systemic effects are likely modulated by microbial metabolites such as short-chain fatty acids (SCFAs), bile acids, and tryptophan catabolites, linking gut dysbiosis to host metabolic disruption [18]. However, despite mounting evidence of compositional microbiome changes during infection, the functional consequences, particularly shifts in microbial gene content, remain poorly understood. Genes encoding carbohydrate-active enzymes (CAZymes), essential for polysaccharide breakdown and host–microbiota interactions, are especially under-characterized. Moreover, regional differences in microbiome structure and function, between the small and large intestines, are rarely examined in this context, limiting insight into the spatial dynamics of infection. Although mice are the predominant model for *T. gondii* studies [19–23], the rat offers distinct advantages: it harbors a more complex and human-relevant microbiota, is commonly used in metabolic research [24], and exhibits resistance to acute toxoplasmosis while more accurately modeling chronic infection [25]. Yet, the effects of *T. gondii* on the rat gut microbiome and metabolome remain largely unexplored [26,27].

These gaps point to a pressing need for more comprehensive, functionally oriented studies of host–parasite–microbiota interactions. We hypothesize that *T. gondii* infection induces stage-specific alterations in both the gut microbiome and systemic metabolome, with distinct consequences during acute and chronic phases. In this study, we use a rat model to investigate the impact of *T. gondii* infection on the structure and functional capacity of the small and large intestinal microbiota, as well as systemic metabolic responses. By integrating high-throughput metagenomic sequencing with untargeted serum metabolomics, we aim to elucidate the molecular links between infection-driven dysbiosis and host metabolic disturbance. The results improved our understanding of parasite–microbiome–host interactions.

## Materials and methods

### Ethics statement

All animal experimental procedures were approved by the Institutional Animal Care and Use Committee of Shanxi Agricultural University (Approval No. SXAU-EAW-2021XM121001) and conducted in compliance with the Biosafety Law of the People’s Republic of China.

### Experimental design and animal grouping

The *T. gondii* PRU strain (genotype II) used in this study was maintained in laboratory Kunming mice. Eighteen Sprague-Dawley rats (8–9 weeks old) were randomly assigned to three groups (*n* = 6 per group): uninfected control (CON), acute infection (AI), and chronic infection (CI). Rats were housed under specific pathogen-free (SPF) conditions with a 12-hour light/dark cycle, controlled temperature (22 ± 2°C), and humidity (50–60%), and were provided with standard laboratory chow and autoclaved water *ad libitum*. Although no formal power calculation was conducted, a sample size of six animals per group was chosen based on previous exploratory multi-omics infection studies, balancing feasibility with statistical robustness. To enhance reliability, strict quality control measures were implemented, and multiple complementary statistical methods and multivariate analyses were applied to identify biologically meaningful differences. Randomization was applied during group allocation, and investigators responsible for sample processing and data analysis were blinded to group assignments to minimize bias.

### Infection protocol and sample collection

Rats in the AI and CI groups were orally inoculated with 3,000 *T. gondii* PRU cysts, while the CON group received an equal volume of phosphate-buffered saline (PBS). The choice of day 7 and day 40 post-infection to represent acute and chronic stages, respectively, was based on established literature [26]. At the designated time points following euthanasia (CON: before infection; AI: day 7 post-infection; CI: day 40 post-infection), at least 2 g of small intestinal contents (a mixture of duodenum, jejunum, and ileum) and large intestinal content (cecum) were collected under sterile conditions and stored at –80°C for DNA extraction. Small intestinal contents were homogenized and analyzed as pooled samples per animal, whereas large intestinal contents were analyzed separately. For non-targeted metabolomics analysis, blood was collected via cardiac puncture immediately after euthanasia. Samples were kept at 4°C for 2–3 hours to allow clotting, then centrifuged at 3,000 × g for 15 min at 4°C. Serum was aliquoted into labeled 1.5 ml microcentrifuge tubes, flash-frozen in liquid nitrogen, and stored at –80°C until analysis.

### Confirmation of infection

To confirm infection, intestinal tissues were collected from the AI group on day 7, brain tissues from the CI group on day 40, and corresponding tissues from the CON group as uninfected controls. Genomic DNA was extracted using the TIANamp Genomic DNA Kit (Tiangen Biotech Co., Ltd., Beijing, China) following the manufacturer’s protocol. Infection was confirmed by PCR amplification of the *T. gondii B1* gene. An initial direct PCR assay was performed using the primer pair (forward: 5′- GGAACTGCATCCGTTCATGAG-3′; reverse: 5′- TCTTTAAAGCGTTCGTGGTC -3′). To improve sensitivity and specificity, a semi-nested PCR was subsequently conducted with a second forward primer (5′-TGCATAGGTTGCAGTCACTG-3′) and the original reverse primer, as described previously [28].

### DNA extraction and sequencing

Genomic DNA was extracted from small intestinal and cecal contents using the TIANGEN Magnetic Soil and Fecal DNA Kit, following the manufacturer’s instructions. DNA integrity was evaluated by 1% agarose gel electrophoresis, and DNA concentration was quantified using a Qubit 3.0 Fluorometer (Invitrogen, USA) with the Qubit DNA Assay Kit. For library preparation, 0.2 μg of DNA per sample was used as input material. Sequencing libraries were constructed using the NEBNext Ultra DNA Library Prep Kit (NEB, USA) according to the manufacturer’s protocol, with unique index barcodes assigned to each sample. Genomic DNA was fragmented to an average insert size of 350 bp via sonication for Illumina sequencing. Shotgun metagenomic sequencing was performed on the Illumina NovaSeq 6000 platform, generating paired-end reads of 150 bp.

### Metagenomic assembly and gene catalog construction

Raw Illumina sequencing data were processed using FASTP (v0.23.0) [29] with the following parameters: -q 20 -u 30 -n 5 -y -Y 30 -l 90 --trim_poly_g. This quality control step removed low-quality bases from read ends and filtered out short or potentially contaminated reads. To remove host-derived sequences, high-quality reads were aligned to the host reference genome (GCF_036323735.1, NCBI) using Bowtie2 (v2.5.0) [30], and any matching reads were discarded. The remaining high-quality, non-host reads were retained for downstream analysis. Sample-specific contigs were assembled with MEGAHIT (v1.2.9) [31], employing the --k-list 21,41,61,81,101,121,141 parameter to optimize assembly across multiple k-mer sizes. Open reading frames (ORFs) were predicted from the assembled contigs using Prodigal (v2.6.3) [32] with the -p meta option tailored for metagenomic data. ORFs shorter than 100 base pairs and incomplete sequences were excluded from further analysis. The remaining ORFs were clustered using the MMseqs2 (v7e284) easy-cluster workflow, applying parameters: --split-mode 2 --split-memory-limit 150G --cov-mode 2 -c 0.9 --min-seq-id 0.95 --cluster-mode 2 --cluster-reassign 1 --kmer-per-seq 200 --kmer-per-seq-scale 0.8. This process generated a non-redundant microbial gene catalog containing 15,230,609 genes.

### Taxonomic and functional annotations

Taxonomic classification of bacterial sequences within the microbial gene catalog was conducted using BLASTn (v2.13.0) [33] against the NCBI-NT database (version 5.0, September 2023; Prokaryotes). For functional annotation of protein-coding genes, sequences were aligned against the Kyoto Encyclopedia of Genes and Genomes (KEGG, version 106.0) [34] and Carbohydrate-Active enZYmes (CAZy, August 2022) [35] databases using DIAMOND (v2.1.8.162) [36] with parameters --min-score 60 --query-cover 50. The highest bit-score hit was selected as the representative alignment for each ORF, providing both taxonomic classification and functional annotation. Gene abundance in each sample was quantified by mapping 20 million high-quality reads to the non-redundant gene catalog using Bowtie2 (v2.4.4) [30]. Raw read counts were normalized to transcripts per kilobase per million mapped reads (TPM). Final abundance profiles were derived from TPM values for genes assigned to bacterial taxa, KEGG orthologs (KOs), and CAZymes.

### Serum metabolite extraction

Serum samples were thawed on ice, and 50 μL aliquots were mixed with 200 μL of methanol/acetonitrile (1:1, v/v) to precipitate proteins. The mixtures were vortexed thoroughly, sonicated at low temperature for 30 minutes, and centrifuged at 12,000 rpm for 10 minutes at 4 °C. The resulting supernatants were dried using a vacuum centrifuge and reconstituted in 100 μL of 50% methanol containing 5 ppm 2-chlorophenylalanine as an internal standard. After a second centrifugation, the final supernatants were transferred to sample vials for liquid chromatography–tandem mass spectrometry (LC-MS/MS) analysis. To monitor instrument stability and ensure data quality, a quality control (QC) sample was prepared by pooling 10–20 μL aliquots from each sample filtrate.

### Chromatographic and mass spectrometry conditions

Chromatographic separation was performed using a Thermo Vanquish ultra-high-performance liquid chromatography (UHPLC) system (Thermo Fisher Scientific, USA) equipped with an ACQUITY UPLC HSS T3 column (2.1 × 100 mm, 1.8 µm; Waters Corporation). The mobile phases consisted of water with 0.1% formic acid (mobile phase A) and acetonitrile with 0.1% formic acid (mobile phase B). The elution gradient was programmed as follows: 0–1 min, 5% B; 1–7 min, linear increase from 5% to 95% B; 7–8 min, held at 95% B; 8–8.1 min, decreased from 95% to 5% B; and 8.1–12 min, maintained at 5% B for column re-equilibration. The flow rate was set to 0.4 mL/min, with the column temperature maintained at 40 °C, the autosampler at 8 °C, and an injection volume of 2 μL [37].

Mass spectrometry analysis was carried out on a Thermo Orbitrap Exploris 120 mass spectrometer operating in data-dependent acquisition (DDA) mode under both positive and negative electrospray ionization (ESI) conditions, controlled by Xcalibur software (v4.7). A heated electrospray ionization (HESI) source was used with the following parameters: spray voltage of +3.5 kV (positive mode) and –3.0 kV (negative mode); sheath gas flow at 40 arbitrary units; auxiliary gas at 15 arbitrary units; capillary temperature at 325 °C; and auxiliary gas heater temperature at 300 °C.

Full MS scans were acquired at a resolution of 60,000 over an m/z range of 100–1000, with the automatic gain control (AGC) target set to standard and a maximum injection time (Max IT) of 100 ms. The four most intense precursor ions per scan were selected for MS/MS fragmentation via higher-energy collisional dissociation (HCD) at a normalized collision energy of 30%, with a resolution of 15,000 and automatic Max IT. Dynamic exclusion was enabled with an 8-second exclusion window to minimize repeated fragmentation of identical ions [38]. All experimental and QC samples were analyzed under consistent chromatographic and mass spectrometric conditions. Prior to sample analysis, 3–5 consecutive QC injections were performed to equilibrate the system. Throughout the analytical run, QC samples were injected every 3–6 samples to monitor instrument stability and ensure data quality.

### Metabolite identification

Raw data files in.raw format were imported into Compound Discoverer 3.3 (Thermo Fisher Scientific) for processing. Peak extraction, alignment, and baseline correction were performed using the software’s enhanced peak detection algorithm and peak quality scoring system. Background interference and low signal-to-noise ratio peaks were effectively suppressed through unique peak scoring and filtering functions. Peaks present in fewer than 50% of QC samples were removed, and a gap-filling algorithm was applied to address missing values. Data normalization was performed using the total peak area method.

Metabolite identification was conducted by integrating multiple online databases, including mzCloud (https://www.mzcloud.org/), LIPID MAPS (https://www.lipidmaps.org/), the Human Metabolome Database (HMDB; https://hmdb.ca/), the MassBank of North America (MoNA; https://mona.fiehnlab.ucdavis.edu/), and the NIST_2020_MSMS spectral library. Mass spectrometry parameters were set as follows: MS1 mass tolerance of 15 ppm and MS2 spectral match threshold of 50. To minimize false positives, metabolite annotation combined retention time, accurate mass (mass error <10 ppm), MS/MS fragmentation spectra, and collision energy data, followed by rigorous manual verification of candidate matches.

Metabolite identification adhered to the Metabolomics Standards Initiative (MSI) guidelines, with all reported metabolites assigned Level 2 confidence or higher [39]. Although authentic standards were not available for all compounds, key metabolites highlighted in this study were stringently confirmed by MS/MS spectral matching. Future targeted metabolomics analyses will validate these critical metabolites using authentic standards.

### Statistical analysis and visualization

Species richness and Shannon diversity indices were calculated based on both taxonomic profiles and gene abundance data. β-diversity was evaluated using principal coordinate analysis (PCoA) based on Bray–Curtis dissimilarity, and group differences were assessed with permutational multivariate analysis of variance (PERMANOVA) using 1,000 permutations. Statistical differences in diversity indices, taxonomic composition, and functional gene abundance between groups were evaluated using the Wilcoxon rank-sum test. To control for multiple comparisons, *P*-values were adjusted using the Benjamini–Hochberg false discovery rate (FDR) method. Pathways, functions, and metabolites with FDR-adjusted *p*-values < 0.05 were considered statistically significant. Rarefaction curves were generated using the vegan package (v2.6.4) [40]. Heatmaps were constructed with the ComplexHeatmap package (v2.15.4) [41]. Principal component analysis (PCA) and partial least squares discriminant analysis (PLS-DA) were performed using the ropls package, with score plots generated to visualize metabolic differences across groups. All additional data visualizations were produced using the ggplot2 package (v4.2.3). All statistical analyses were performed in R (v4.4.3).

## Results

### Construction of a non-redundant gene library

To investigate the impact of T. gondii infection on the intestinal microbiome of rats, metagenomic sequencing was conducted on intestinal contents from 18 Sprague-Dawley rats. After rigorous quality filtering and removal of host-derived sequences, 148 Gb of high-quality clean reads were obtained. De novo assembly generated 14,185,162 scaffolds with an N50 of 1,187 bp, reflecting good assembly continuity. Gene prediction identified 5,733,102 non-redundant microbial genes, with an average gene length of 452.75 bp. The resulting gene catalog exhibited robust assembly quality, supported by strong N50 and N90 metrics, and provides a comprehensive reference for subsequent functional and taxonomic analyses.

### Effect of *T. gondii* infection on rat intestinal microbiota

**Fig 1:**
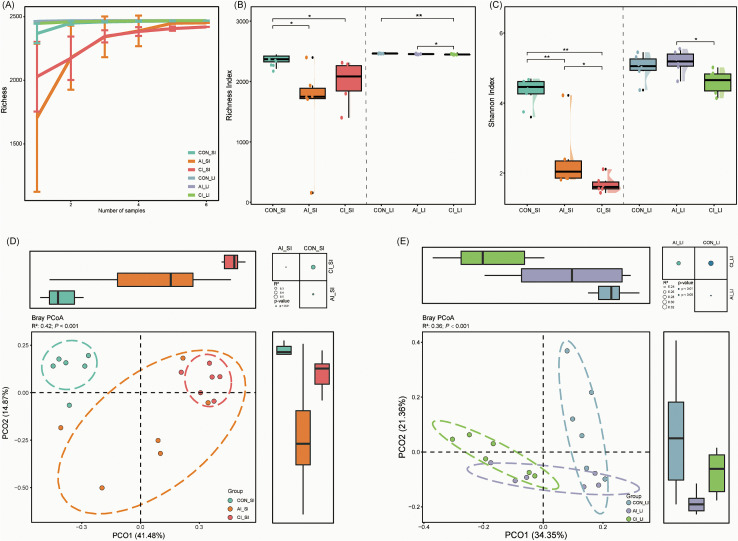
Effects of *T. gondii* infection on rat gut microbiota diversity. **(A)** Rarefaction curves showing the relationship between gene richness and sequencing depth, indicating that microbial gene diversity reaches a plateau with increased sequencing effort. **(B–C)** Raincloud plots depicting richness and Shannon diversity index in *T. gondii*-infected and uninfected rats. Individual sample values are shown as dots, density plots represent the distribution, and box plots summarize the median and interquartile range. Statistical significance was determined using the Wilcoxon rank-sum test: *P < 0.05; **P < 0.01; ***P < 0.001. **(D–E)** Principal coordinate analysis (PCoA) based on Bray–Curtis distances depicting differences in microbial community composition across infection stages. Ellipses represent 95% confidence intervals. Box plots adjacent to PCoA axes display sample scores for PCoA1 and PCoA2, with boxes indicating medians and interquartile ranges, and whiskers extending to 1.5 × the IQR. Overall and pairwise differences in community structure were assessed using PERMANOVA, with effect sizes shown in the upper right scatter plot. *P*-values were calculated using the adonis test with 1,000 permutations. LI: large intestine; SI: small intestine.

To investigate the effect of *T. gondii* infection on the rat intestinal microbiome, the non-redundant gene catalog was compared against the NCBI-NT database using BLASTn, resulting in the taxonomic assignments of 3,720,400 bacterial genes. Rarefaction curves indicated that bacterial richness approached saturation with increasing sample size, confirming sufficient sequencing depth for comprehensive microbial profiling (Fig 1A). Alpha diversity analysis revealed a significant reduction in both microbial richness and Shannon diversity in the small intestine during acute and chronic infection. Richness decreased from 2,358 ± 86.96 in controls to 2,455.5 ± 6.5 in acute infection (fold change [FC] = 0.69, *P* < 0.05) and 1,994.5 ± 351.10 in chronic infection (FC = 0.85, *P* < 0.05). Similarly, Shannon diversity decreased from 4.35 ± 0.40 in controls to 2.39 ± 0.92 in acute infection (FC = 0.55, *P* < 0.01) and 1.68 ± 0.24 in chronic infection (FC = 0.39, *P* < 0.01).

In the large intestine, microbial richness remained largely stable during acute infection but was significantly reduced during chronic infection (control: 2,463 ± 2.80; acute: 2,455 ± 6.50; chronic: 2,448 ± 5.64; *P* < 0.05). However, Shannon diversity did not differ significantly between control and acute groups (5.03 ± 0.38 vs. 5.17 ± 0.33), but declined in the chronic group (4.60 ± 0.34). Comparisons between infection stages further revealed reduced richness (FC = 1.00, *P* < 0.05) and Shannon diversity (FC = 1.12, *P* < 0.05) in chronic relative to acute infection (Fig 1B–C).

Beta diversity analysis using PCoA of Bray–Curtis dissimilarity demonstrated pronounced shifts in microbial community composition in both the small intestine (PERMANOVA, R2 = 0.42, *P* < 0.001) and large intestine (PERMANOVA, R2 = 0.36, *P* < 0.001) (Fig 1D–E), underscoring the substantial impact of *T. gondii* infection on gut microbial structure.

### Taxonomic analysis of the rat intestinal microbiome following *T. gondii* infection

**Fig 2:**
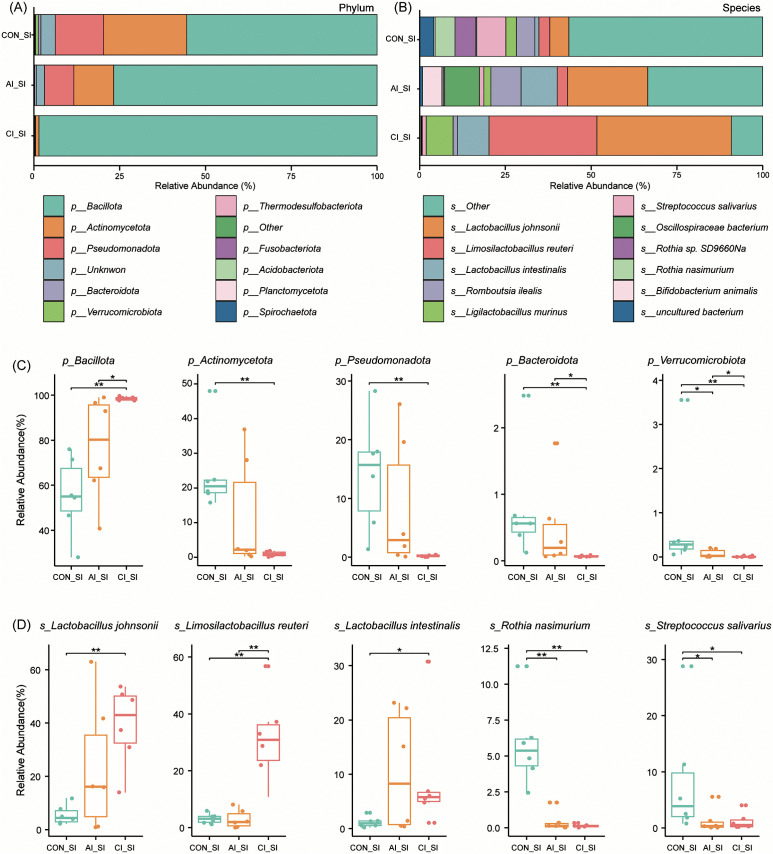
Composition of intestinal microbial communities in the small intestine of rats before and after *T. gondii* infection. **(A–B)** Bar plots showing taxonomic composition at the phylum and species levels across groups before and after infection. **(C)** Box plots depicting the relative abundance of major bacterial phyla, including *Bacillota*, *Actinomycetota*, *Pseudomonadota*, *Bacteroidota*, and *Verrucomicrobiota*, at different infection stages. **(D)** Box plots illustrating changes in the relative abundance of representative bacterial species, including *Lactobacillus johnsonii*, *Limosilactobacillus reuteri*, *Lactobacillus intestinalis*, *Rothia nasimurium*, and *Streptococcus salivarius*, following *T. gondii* infection. Statistical significance was determined using the Wilcoxon rank-sum test: *P < 0.05; **P < 0.01.

To investigate the taxonomic impact of *T. gondii* infection, we profiled microbial communities in the small and large intestines of rats. PCoA confirmed significant shifts in microbial composition in both intestinal regions after infection. To identify taxa contributing to these changes, we performed comparative analyses at both the phylum and species levels, revealing distinct alterations across acute and chronic stages. In the small intestine, the dominant phyla were *Bacillota*, *Actinomycetota*, and *Pseudomonadota* (Fig 2A). The relative abundance of *Bacillota* increased progressively post-infection, reaching significantly higher levels during chronic infection compared with both controls (*P* < 0.01) and the acute stage (*P* < 0.05). Conversely, *Actinomycetota*, *Pseudomonadota*, *Bacteroidota*, and *Verrucomicrobiota* declined over the course of infection, with abundances in the chronic stage significantly lower than in controls (*P* < 0.01) (Fig 2C). At the species level, the five most abundant taxa were *Lactobacillus johnsonii*, *Limosilactobacillus reuteri*, *Lactobacillus intestinalis*, *Romboutsia ilealis*, and *Ligilactobacillus murinus* (Fig 2B). Differential abundance analysis revealed that *L. johnsonii* (*P* < 0.01), *L. reuteri* (*P* < 0.01), and *L. intestinalis* (*P* < 0.05) were significantly enriched during chronic infection. In contrast, *Rothia nasimurium* (*P* < 0.01) and *Streptococcus salivarius* (*P* < 0.05) were significantly depleted in both acute and chronic stages (Fig 2D).

**Fig 3:**
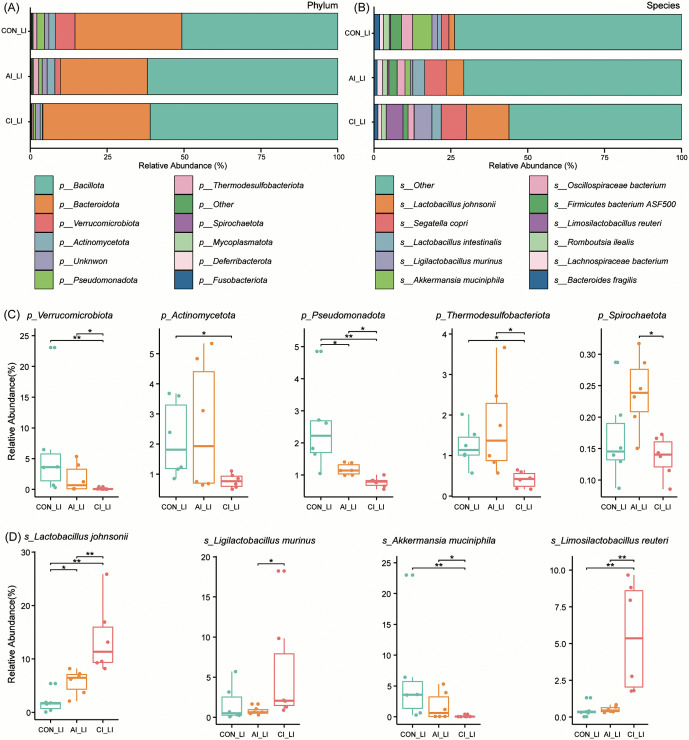
Composition of intestinal microbial communities in the large intestine of rats before and after *T. gondii* infection. **(A–B)** Bar plots illustrating taxonomic composition at the phylum and species levels across different groups before and after infection. **(C)** Box plots depicting the relative abundance of major bacterial phyla, including *Verrucomicrobiota*, *Actinomycetota*, *Pseudomonadota*, *Thermodesulfobacteriota* and *Spirochaetota*, at different infection stages. **(D)** Box plots illustrating changes in the relative abundance of representative bacterial species, including *Lactobacillus johnsonii*, *Liqilactobacillus murinus*, *Akkermansia muciniphila* and *Limosilactobacillus reuteri*, following *T. gondii* infection. Statistical significance was assessed using the Wilcoxon rank-sum test: * *P* < 0.05; **P < 0.01.

In the large intestine, the dominant phyla were *Bacillota*, *Bacteroidota*, and *Verrucomicrobiota* (Fig 3A). The relative abundance of *Verrucomicrobiota* decreased progressively with infection, reaching significantly lower levels during chronic infection compared with controls (*P* < 0.01) and acute infection (*P* < 0.05). *Actinomycetota* abundance was also significantly reduced in the chronic stage (*P* < 0.05). Similarly, *Pseudomonadota* levels were consistently lower than controls in both acute and chronic stages (*P* < 0.05). *Thermodesulfobacteriota* was significantly reduced in the chronic group compared with both controls and acute infection (*P* < 0.05), while *Spirochaetota* was significantly decreased during the chronic stage relative to acute infection (*P* < 0.05) (Fig 3C). At the species level, the five most abundant taxa were *L. johnsonii*, *Segatella copri*, *L. intestinalis*, *L. murinus*, and *Akkermansia muciniphila* (Fig 3B). *L. johnsonii* was significantly enriched in both acute (*P* < 0.05) and chronic (*P* < 0.01) stages compared with controls, while *L. murinus* was more abundant during chronic than acute infection (*P* < 0.05). In contrast, *A. muciniphila* exhibited a significant decline in the chronic stage compared with both controls (*P* < 0.05) and acute infection (*P* < 0.01). Importantly, *L. reuteri* was markedly enriched during chronic infection relative to both control and acute groups (*P* < 0.01) (Fig 3D).

### Functional analysis of intestinal bacterial communities following *T. gondii* infection

**Fig 4:**
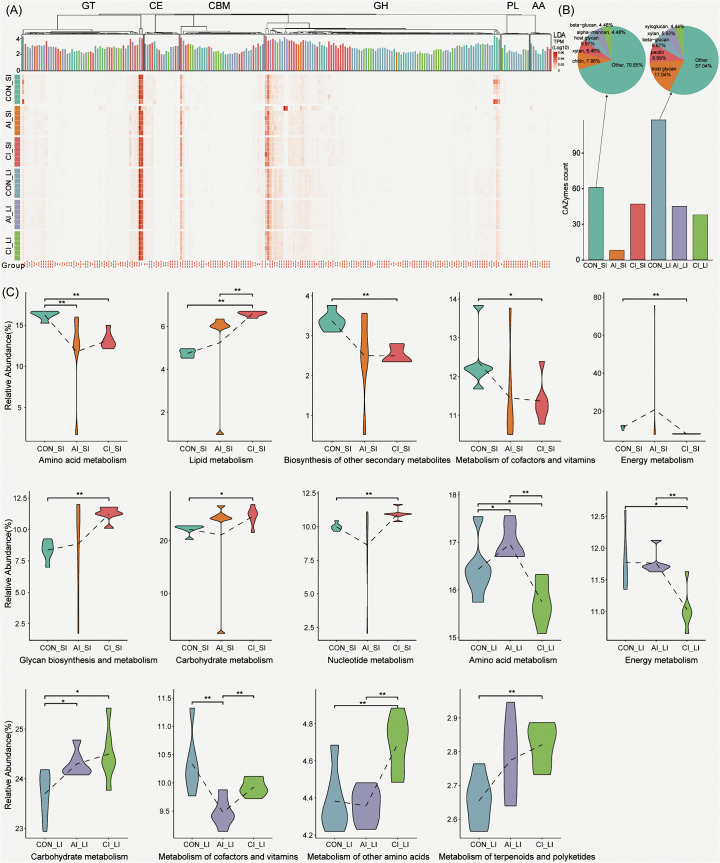
Functional analysis of the intestinal microbial community following *T. gondii* infection. **(A)** Heatmap of log₁₀(TPM + 1) transformed abundances of CAZymes significantly enriched by LEfSe (Linear Discriminant Analysis (LDA) score > 2). Rows represent samples, grouped by treatment and intestinal segment, while columns denote CAZy families grouped by class. Asterisks (*) indicate a CAZy families with significant differences (LDA score > 2). The top annotation bar plot shows the magnitude and direction of enrichment, colored by the enriched group. **(B)** Bar plots significantly enriched CAZymes in each group. Pie charts illustrate predicted substrate-type proportions for control-enriched CAZymes in the small and large intestine. **(C)** Violin plots representative metabolic pathways in the small and large intestine across infection stages. Statistical significance was assessed by the Wilcoxon rank-sum test: *P < 0.05; **P < 0.01.

To assess the functional consequences of *T. gondii* infection on the gut microbiota, we annotated bacterial gene content against the CAZy and KEGG databases, focusing on CAZymes and metabolic pathways in both the small and large intestines during acute and chronic infection. CAZy analysis showed that 18.23% (678,168 of 3,720,400) of predicted protein-coding genes encoded at least one CAZyme, including 18 auxiliary activities (AAs), 75 carbohydrate-binding modules (CBMs), 19 carbohydrate esterases (CEs), 289 glycoside hydrolases (GHs), 88 glycosyltransferases (GTs), and 63 polysaccharide lyases (PLs) (Fig 4A). Before infection, LEfSe analysis revealed distinct regional CAZyme profiles: in the small intestine, enzymes were enriched for chitin, xylan, and host glycans, whereas in the large intestine, CAZymes predominantly targeted host glycans, pectin, and β-glucans (Fig 4B). These differences likely reflect the nutrient landscape of each region, with the small intestine exposed to host-derived glycans and partially digested substrates, and the large intestine enriched in microbes specialized in degrading complex polysaccharides [42,43]. Following *T. gondii* infection, CAZyme abundance declined significantly in both intestinal regions, suggesting impaired microbial carbohydrate metabolism.

KEGG annotation assigned 41.45% (1,542,269 of 3,720,400) of genes to 7,016 unique KOs, grouped into 405 pathways. Genes involved in metabolic processes were the most abundant (S2 Fig), highlighting the broad functional influence of infection. Differential analysis revealed region- and stage-specific alterations. In the small intestine, amino acid metabolism was significantly reduced during both acute and chronic infection (*P* < 0.01), whereas lipid metabolism increased (*P* < 0.01). In the chronic stage, pathways related to the biosynthesis of secondary metabolites (*P* < 0.01), cofactors and vitamins (*P* < 0.05), and energy metabolism (*P* < 0.05) were downregulated, while glycan biosynthesis and metabolism (*P* < 0.01), carbohydrate metabolism (*P* < 0.05), and nucleotide metabolism (*P* < 0.01) were significantly upregulated. In the large intestine, amino acid metabolism was elevated during acute infection but declined sharply in the chronic stage (*P* < 0.05). Energy metabolism was significantly suppressed during chronic infection (*P* < 0.05), while carbohydrate metabolism increased in both acute and chronic stages (*P* < 0.05). Cofactor and vitamin metabolism decreased during acute infection (*P* < 0.01), whereas pathways related to other amino acids and terpenoids/polyketides were significantly enriched during chronic infection (*P* < 0.01) (Fig 4C). These findings demonstrate that *T. gondii* infection induces profound disruptions in microbial functional capacity, altering carbohydrate utilization and metabolic pathways in a region- and stage-specific manner, with potential consequences for host nutrient metabolism and immune regulation.

### Metabolic profile analysis of serum using untargeted metabolomics

To assess whether alterations in the gut microbiota of *T. gondii*-infected rats translate into systemic metabolic changes, we performed untargeted serum metabolomics on samples collected before and after infection.QC was rigorously maintained throughout LC-MS/MS analysis. TICs from QC injections exhibited highly consistent retention times and peak intensities across both positive and negative ionization modes. Pearson correlation analysis confirmed strong reproducibility (r > 0.95), while relative standard deviation (RSD) analysis showed that over 75% of characteristic peaks in QC samples had RSD values <30%. Together, these metrics validate the robustness of the LC-MS platform and the reliability of the metabolomic data (S3 Fig).

**Fig 5:**
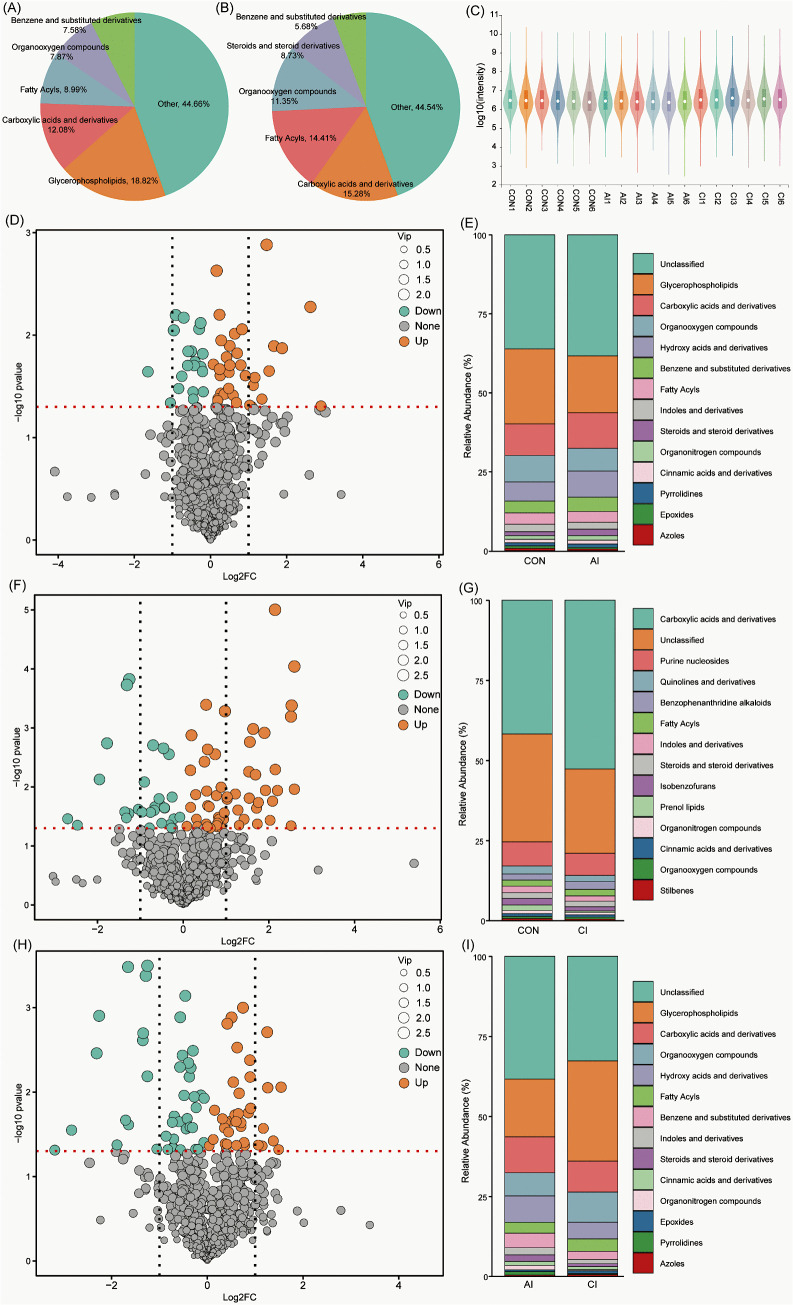
Untargeted serum metabolomics analysis before and after *T. gondii* infection. **(A–B)** Pie charts illustrating the classification of metabolites detected in rat serum under positive and negative ionization modes. **(C)** Violin plot of metabolite expression distributions across samples. The central line denotes the median; box edges indicate the 25th and 75th percentiles; whiskers extend to the 10th and 90th percentiles. The outer shapes represent the kernel density estimation. **(D–I)** Volcano plots and bar charts of differential serum metabolites between groups: **(D–E)** control (CON) vs. acute infection (AI); **(F–G)** CON vs. chronic infection (CI); **(H–I)** AI vs. CI. Volcano plots display log2-transformed fold change on the x-axis and –log₁₀ *P*-value (Student’s *t*-test) on the y-axis. Vertical dashed lines mark |log_2_FC| = 1 (twofold change), and the horizontal red line marks *P* = 0.05. Point colors indicate significance, while the point size corresponds to the variable importance in projection (VIP) score from OPLS-DA.

Untargeted profiling identified 490 metabolites in positive and 393 in negative ion mode. Annotation using KEGG, HMDB, and LipidMaps indicated that the serum metabolome was enriched in carboxylic acids and derivatives, glycerophospholipids, fatty acyls, and organooxygen compounds (Fig 5A–B). Density distribution plots further confirmed the consistency of global abundance patterns across samples (Fig 5C). Differential metabolites were defined by fold change (FC > 1), *t*-*t*est significance (*P* < 0.05), and variable importance in projection (VIP > 1) from the OPLS-DA model. Score plots revealed clear group separation, underscoring robust classification (S4 Fig). Between the CON and AI groups, 53 differential metabolites were detected, predominantly glycerophospholipids, carboxylic acids and derivatives, and organooxygen compounds (Fig 5D–E). The CON vs. CI comparison revealed 84 differential metabolites, mainly carboxylic acids and derivatives, purine nucleosides, and quinoline derivatives (Fig 5F–G). Finally, 80 metabolites differentiated the AI and CI groups, with the majority belonging to glycerophospholipids, carboxylic acids and derivatives, and organooxygen compounds (Fig 5H–I).

### Pathway enrichment analysis of differential metabolites

**Fig 6:**
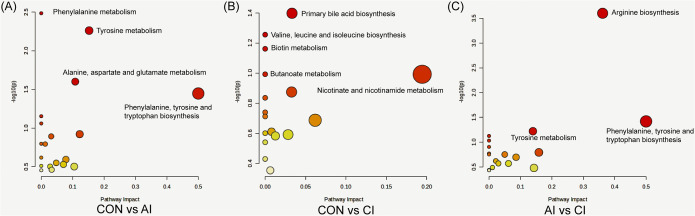
KEGG pathway enrichment analysis of differential serum metabolites. Each dot represents a metabolic pathway. The x-axis shows the pathway impact score, while the y-axis indicates–log₁₀ (*P* value). Dot size reflects the impact score, and the color gradient from yellow to red corresponds to increasing statistical significance. Results are shown for **(A)** control (CON) vs. acute infection (AI), **(B)** CON vs. chronic infection (CI), and **(C)** AI vs. CI.

To elucidate the biological significance of the observed metabolite changes, pathway enrichment analysis was conducted using the MetaboAnalyst platform (https://www.metaboanalyst.ca/, accessed May 30, 2025). Pathways were ranked according to *P*-values and pathway impact scores (S2 Table). In the CON vs. AI comparison (Fig 6A), significantly enriched pathways included phenylalanine metabolism, tyrosine metabolism, and alanine, aspartate, and glutamate metabolism. In the CON vs. CI comparison (Fig 6B), primary bile acid biosynthesis, valine/leucine/isoleucine biosynthesis, and biotin metabolism were enriched. For the AI vs. CI comparison (Fig 6C), enrichment was observed in arginine biosynthesis, tyrosine metabolism, and the combined biosynthetic pathway of phenylalanine, tyrosine, and tryptophan.

### Correlation between gut microbial species and differential metabolites

**Fig 7:**
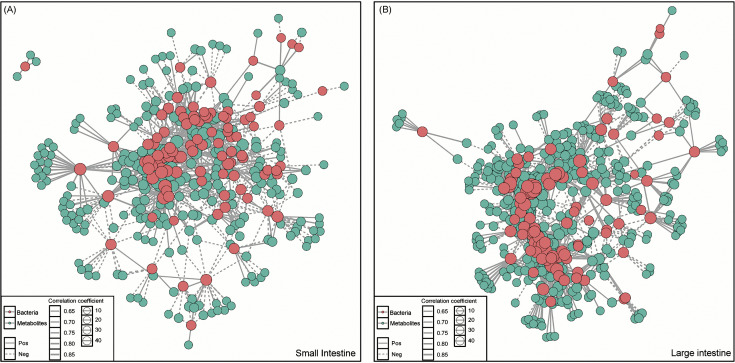
Correlation network between intestinal bacterial species and differential serum metabolites. Species–metabolite associations are shown at the species level. Solid lines indicate positive correlations, and dashed lines indicate negative correlations. Edge thickness reflects the correlation strength (Spearman’s correlation coefficient), while node size denotes connectivity (number of associations). Results are shown for **(A)** the small intestine. **(B)** the large intestine.

To explore microbial contributions to systemic metabolic alterations, Spearman correlation analysis was performed between differential serum metabolites and gut microbial species (ρ > 0.6, *P* < 0.01). This revealed a complex network of associations, with distinct bacterial taxa linked to specific metabolite profiles. In the small intestine, *L. johnsonii* correlated positively with sulfuric acid 4-methoxyphenyl ester, 1,2-dihydronaphthalene-1,2-diol, and 1-deoxy-D-altro-heptulose 7-phosphate, but negatively with *p*-cresol, L-glutamine, and indole-3-glycol aldehyde. *L.s reuteri* was positively associated with pramipexole, sulfuric acid 4-methoxyphenyl ester, and 1,2-dihydronaphthalene-1,2-diol, but negatively correlated with *p*-cresol, succinic acid, and 2-hydroxy-3-methylbenzalpyruvate. *L. intestinalis* correlated positively with catechol and negatively with L-lysine and N6,N6,N6-trimethyl-L-lysine. Conversely, *Rothia nasimurium* was positively associated with L-lysine and N6,N6,N6-trimethyl-L-lysine, but negatively with 4’-hydroxy-5,6,7,8-tetramethoxyflavone and hydroxyspirilloxanthin. *Streptococcus salivarius* showed positive correlations with sphinganine and negative correlations with 16α-hydroxyandrost-4-ene-3,17-dione (Fig 7A; S3 Table).

In the large intestine, *Lactobacillus johnsonii* correlated positively with catechol, 3,4-dihydroxyphenylglycol, and 8-methylthiooctanaldoxime, but negatively with *p*-cresol, succinic acid, and valienone. *Ligilactobacillus murinus* showed positive correlations with bromobenzene-3,4-oxide, 3-keto-β-D-galactose, and 4-hydroxy-3-prenylbenzoate, and negative correlations with *p*-cresol, L-valine, and 2-hydroxyethanesulfonate. *A. muciniphila* was positively associated with *p*-cresol, succinic acid, and 2-hydroxy-3-methylbenzalpyruvate, but negatively correlated with L-glyceric acid, catechol, and quinoline-3,4-diol. Additionally, *L. reuteri* correlated positively with dodecanoic acid and negatively with monoisobutyl phthalic acid, N-(3,4-dichlorophenyl) malonamic acid, and N6-cis-*p*-coumaroylserotonin (Fig 7B; S3 Table).

## Discussion

This study provides a comprehensive characterization of how *T. gondii* infection reshapes the rat intestinal microbiome and systemic metabolism. Using high-throughput metagenomic sequencing, we constructed a high-quality, non-redundant gene catalog that revealed significant alterations in microbial diversity, taxonomic structure, and functional capacity. These microbiome changes were paralleled by distinct shifts in systemic metabolite profiles, highlighting the complex, multidirectional interplay between host, microbe and *T. gondii* during infection.

Consistent with reports in rats [26], mice [19–23], and humans [44], *T. gondii* infection significantly reduced microbial diversity and induced compositional alterations during acute and chronic stages, with the small intestine showing particularly marked changes. Detailed taxonomic profiling revealed region-specific disruptions: in the small intestine, there was a progressive expansion of *Bacillota* and enrichment of *Lactobacillus* species during chronic infection, potentially reflecting a compensatory microbial response to parasite-driven alterations in the intestinal environment. Conversely, broad reductions in *Actinomycetota*, *Pseudomonadota*, *Bacteroidota*, and *Verrucomicrobiota*, particularly *A. muciniphila*, suggest widespread dysregulation of microbial equilibrium, impaired gut barrier function, and diminished mucin-degradation capacity. The large intestine mirrored these trends, with marked decreases in beneficial taxa such as *A. muciniphila*, critical for maintaining mucosal integrity. Simultaneously, enrichment of *L. johnsonii* and *L. reuteri* suggests these species may play a role in modulating host immunity during infection. This progressive loss of diversity reflects a gut ecosystem that becomes increasingly unstable, with reduced resilience and heightened susceptibility to secondary infections and immune dysfunction [45]. Future studies should aim to determine whether enrichment of *Lactobacillus spp*. and depletion of *A. muciniphila* causally contribute to immune imbalance and disease progression. Integrating microbiome, metabolome, and targeted probiotic or metabolite interventions will be critical to clarify their mechanistic roles in *T. gondii* infection.

Our findings contrast with previous rat studies reporting no significant differences in microbial alpha or beta diversity between *T. gondii*-infected and control animals during the chronic phase, with cecal microbial richness, diversity, and structure largely unchanged [27]. These findings indicate that gut dysbiosis may not consistently manifest during *T. gondii* infection, especially in the chronic stage However, the divergence between these results and ours points to factors that may shape distinct outcomes within the same host species. Anatomical sampling appears critical. While prior work focused on the caecum, we observed the strongest microbial alterations in the small intestine, with moderate effects in the large intestine, reflecting the proximal gut’s susceptibility to *T. gondii*-associated pathology during acute infection. Thus, apparent stability in the caecum may reflect regional heterogeneity in host–parasite–microbiota interactions rather than true absence of dysbiosis. Infection stage is also likely influential. Previous analyses examined only the chronic phase [27], whereas we assessed both acute and chronic stages. Dysbiosis may primarily arise from acute immunopathology, with partial recovery or re-equilibration by the chronic phase, especially in the caecum. Additional variables, including parasite strain (PRU, genotype II vs. others), inoculum type and dose, host background, diet, housing, sequencing depth, and analytical pipelines, may further contribute. These factors underscore the importance of interpreting microbiome studies within their specific experimental context.

A previous metagenomic study in *T. gondii*-infected mice reported substantial reductions in CAZy families and widespread alterations in KEGG pathways, including LPS biosynthesis, carbohydrate metabolism, and other key microbial functions [45]. Consistently, our metagenomic analyses in rats revealed a reduction in CAZyme abundance, indicating impaired microbial capacity to degrade dietary polysaccharides into fermentable substrates. CAZymes are essential for producing SCFAs, which support energy supply, epithelial barrier integrity, and immune homeostasis. Consequently, depletion of CAZymes may reduce SCFA production, potentially compromising gut barrier function and exacerbating immune dysregulation during infection [46,47]. Interestingly, metabolic profiling of the small intestine showed upregulated carbohydrate metabolism during both acute and chronic infection. This apparent discrepancy likely reflects compensatory mechanisms: while overall CAZyme activity declines, microbial community shifts may favor taxa with enhanced or alternative carbohydrate utilization, allowing the gut ecosystem to sustain energy production under infection-induced stress.

Metabolic profiling revealed stage- and region-specific patterns in amino acid and lipid metabolism. In the small intestine, amino acid metabolism was suppressed during both acute and chronic infection, likely reflecting microbial adaptation to nutrient shifts and local inflammatory stress [45]. In contrast, in the large intestine, amino acid metabolism initially increased during acute infection but declined sharply in the chronic phase, underscoring region-specific effects of infection, likely driven by differences in microbial composition, nutrient availability, and local immune responses. Small intestinal lipid metabolism pathways were upregulated across infection stages. While *T. gondii* actively scavenges host lipids to support intracellular replication and membrane biogenesis [48,49], reducing luminal lipid availability, this scarcity may itself drive microbial adaptation. Certain bacteria respond to limited lipid availability by enhancing lipid utilization for energy production, membrane remodeling, or stress resilience. Thus, the observed increase in microbial lipid metabolism likely represents a compensatory functional response rather than an absolute increase in luminal lipids, contributing to the progressive enhancement of lipid metabolic pathways observed in chronic infection. Other prominent changes during chronic infection in the large intestine included suppressed energy metabolism alongside enhanced metabolism of terpenoids, polyketides, and secondary metabolites, compounds known to modulate host immunity or mediate microbial competition, for example, colibactin, which promotes epithelial damage and inflammation [50].

Serum metabolomics mirrored these gut alterations, reflecting systemic consequences of infection. Serum from infected rats showed extensive metabolic shifts, particularly in amino acid, bile acid, and aromatic compound pathways. Key perturbations included elevated carboxylic acids, glycerophospholipids, fatty acyls, purine nucleosides, and quinoline derivatives, all linked to inflammation, immune regulation, and energy metabolism. These findings are consistent with prior serum metabolomic studies in BALB/c mice, which reported disturbances in amino acids (e.g., tryptophan, kynurenine), lipids, nucleotides, and aromatic compounds [51]. Reductions in taurocholic acid and disruptions in primary bile acid biosynthesis were also observed during both acute and chronic stages, aligning with altered lipid and energy metabolism in the gut [52]. These results highlight coordinated gut and systemic metabolic reprogramming during *T. gondii* infection.

Spearman correlation analyses further underscored the microbiome’s influence on host metabolism. In the small intestine, *L. johnsonii* and *L. reuteri* were positively associated with phenolic and sulfur-containing metabolites, suggesting potential roles in detoxification and immune modulation. This aligns with prior evidence that *L. reuteri* can metabolize tryptophan into indole derivatives, regulating mucosal immunity via aryl hydrocarbon receptor signaling [53]. These taxa were also negatively associated with inflammatory metabolites such as p-cresol and succinic acid, implicated in intestinal barrier dysfunction and oxidative stress. These associations raise the possibility that supplementation with *L. johnsonii* or *L. reuteri* could help restore microbial balance and modulate immunity during chronic infection, although this requires experimental validation [54]. In the large intestine, similar patterns were observed for *L. johnsonii*, *L. murinus*, and *A. muciniphila*. Interestingly, *A. muciniphila* correlated strongly with metabolites involved in energy and amino acid metabolism, consistent with previous reports linking this taxon to circulating acylcarnitines, ketone bodies, and aromatic amino acid intermediates under metabolic stress [55]. *A. muciniphila* has also been inversely associated with liver injury markers and phenolic sulfates [56]. These findings indicate that functional shifts in the gut microbiota reflect not only compositional changes, but also systemic metabolite shifts with consequences for nutrient availability, immune modulation, and metabolic homeostasis.

Pathway enrichment analyses revealed stage-specific metabolic signatures. In the acute stage, phenylalanine, tyrosine, and glutamate metabolism were enriched, reflecting pathways critical for neurotransmission and immune signaling, consistent with findings from previous hepatic and serum metabolomics studies [52,57]. The chronic phase was marked by increased activity in primary bile acid biosynthesis and branched-chain amino acid metabolism, reflecting longer-term disruptions in lipid processing, bile secretion, and energy homeostasis [52]. Additional alterations in arachidonic acid and unsaturated fatty acid metabolism indicate cumulative metabolic stress as infection persists. These stage-specific changes highlight potential biomarkers and therapeutic targets, including neurotransmitter-related amino acids and bile acid signaling. From a therapeutic perspective, microbiota-targeted interventions, such as probiotics, prebiotics, or administration of metabolites promoting beneficial taxa, may complement conventional treatments by enhancing host tolerance and resistance to infection [58]. While promising, these approaches remain speculative and require rigorous experimental validation. Importantly, these systemic metabolite changes paralleled microbial functional alterations identified in the metagenomic data. Suppression of amino acid metabolism in serum was consistent with reduced microbial genes involved in amino acid biosynthesis, suggesting that infection-driven microbial disruption contributes to systemic amino acid deficiency. Conversely, the enrichment of lipid metabolism during chronic infection coincided with microbial taxa enriched for lipid-utilizing capacity, indicating that both the parasite and the altered gut microbiota may jointly drive host lipid metabolic reprogramming.

Some limitations should be acknowledged. Although the rat model harbors a more complex and human-relevant gut microbiota than mice, it does not replicate human toxoplasmosis, particularly regarding clinical outcomes and chronic infection dynamics. Further validation in definitive hosts, such as cats, and clinical studies in humans will be necessary to establish translational relevance. Although the relatively small sample size may limit statistical power, the experimental design aligns with exploratory multi-omics studies, and robustness was enhanced through quality control measures and complementary statistical approaches. Finally, correlations between specific microbes and circulating metabolites should be interpreted as hypothesis-generating rather than evidence of causality. Future work using gnotobiotic rat models, targeted supplementation experiments, or microbial manipulation studies will be necessary to determine whether specific taxa directly produce or mediate the observed metabolic changes.

## Conclusion

This study highlights the central role of the gut microbiota in shaping host metabolic and immune responses during *T. gondii* infection. By integrating metagenomic and metabolomic analyses, we uncovered extensive alterations in microbial composition, functional capacity and associated systemic metabolites. The enrichment of immunomodulatory *Lactobacillus* species alongside the depletion of beneficial taxa such as *A. muciniphila* reflects a pronounced microbial imbalance that may influence disease progression. These taxonomic shifts were accompanied by changes in carbohydrate-active enzymes and metabolic pathways, particularly those involved in amino acid and bile acid metabolism. Correlations between specific microbial taxa and circulating metabolites further underscore the microbiota’s role in maintaining host metabolic homeostasis. Our findings provide a systems-level perspective on host–microbiota–parasite interactions and point to microbiome-targeted interventions as promising therapeutic strategies for managing toxoplasmosis.

## Supporting information

S1 Fig Graphical abstract. Overview of the experimental design and major findings. Eighteen Sprague–Dawley rats were randomly divided into three groups: uninfected control (CON), acute infection (AI, day 7 post-infection), and chronic infection (CI, day 40 post-infection). Rats in the AI and CI groups were orally inoculated with 3,000 *T. gondii* PRU cysts, while controls received PBS. Small and large intestinal contents were collected for metagenomic sequencing, and serum was analyzed by untargeted metabolomics. The integrated multi-omics analysis revealed stage-specific alterations in gut microbial diversity, taxonomic composition, and functional potential, along with systemic metabolic perturbations. Spearman correlation analysis demonstrated coordinated associations between differential microbes and metabolites, illustrating the interaction network among *T. gondii*, the gut microbiota, and host metabolism. Created in BioRender. Jx, Z. (2025) https://BioRender.com/2rh0rbd.
(TIF)

S2 Fig Heatmap of enriched KEGG pathways across samples, illustrating variations in pathway abundance. (TIF)

S3 Fig Quality control assessment of serum metabolomics data. **(A–B)** Total Ion Chromatograms (TICs) for serum samples in positive and negative ion modes. The horizontal axis represents the retention time and the vertical axis shows peak intensity. **(C–D)** QC correlation analysis among QC samples. Each point represents a detected ion (metabolite), with both axes showing the log-transformed signal intensities across different QC replicates. **(E–F)** Distribution of relative standard deviation (RSD) values in QC samples, with the horizontal axis representing RSD and the vertical axis showing the proportion of peaks at each RSD.
(TIF)

S4 Fig Orthogonal partial least squares-discriminant analysis (OPLS-DA) of serum metabolomics data. R^2^X and R^2^Y indicate the variance explained by the X (metabolite quantification) and Y (sample grouping) matrices, respectively. Q^2^ represents the predictive accuracy of the model, reflecting its ability to correctly classify sample groups based on metabolic profiles.
(TIF)

S1 Table Differential metabolite results, listing metabolites with significant changes across treatment groups, including fold changes, and *p*-values. (XLSX)

S2 Table Pathway enrichment analysis of differential metabolites, highlighting significantly impacted metabolic pathways and associated enrichment statistics based on KEGG or other pathway databases. (XLSX)

S3 Table Spearman correlation analysis between gut microbial taxa and circulating metabolites, detailing correlation coefficients, significance values, and taxon–metabolite pairings indicative of microbiome–host metabolic interactions. (XLSX)
